# Exercise in the Treatment of Youth Substance Use Disorders: Review and Recommendations

**DOI:** 10.3389/fpsyg.2017.01839

**Published:** 2017-10-17

**Authors:** Alissa More, Ben Jackson, James A. Dimmock, Ashleigh L. Thornton, Allan Colthart, Bonnie J. Furzer

**Affiliations:** ^1^School of Human Sciences (Exercise & Sport Science), The University of Western Australia, Perth, WA, Australia; ^2^Drug and Alcohol Youth Service, Mental Health Commission and Mission Australia, Perth, WA, Australia

**Keywords:** adolescence, alcohol, drugs, physical activity, recovery, substance misuse

## Abstract

Substance use disorders among youth represent a significant public health concern. It is well established that regular exercise provides important physical and mental health benefits; however, evidence for the role of exercise as an adjunct component within substance use disorder treatment is scarce. In this review, we identify factors associated with the development and persistence of substance use disorders among youth, identify current treatment modalities, and present evidence to support the efficacy of incorporating exercise participation during rehabilitation. We also provide a series of recommendations for future research that explores the feasibility and effectiveness of exercise participation as a complement to substance use disorder treatment among youth.

## Introduction

Adolescence is characterized by rapid physical and psychological transition (Evans et al., 2005), as well as heightened experimentation and risk-taking behavior (e.g., illicit drug and alcohol use) that may underpin short- and longer-term health problems (Loxley et al., 2004). One such problem is the development of substance use disorders (SUDs), which occur when the recurrent use of a substance, namely alcohol and/or drugs, causes clinically and functionally significant impairments, such as health problems, disability, and failure to meet major responsibilities at work, school, or home (American Psychological Association, 2013). Consistent with the fifth edition of the Diagnostic Statistical Manual for Mental Disorders, the term SUD is used in this review to describe the wide range of substance-related disorders that can range from a mild form to severe or chronic conditions, and which historically or colloquially have also been referred to as ‘addiction’ (American Psychological Association, 2013). SUD diagnosis is based on evidence of impaired control, social impairment, risky use, and pharmacological criteria (American Psychological Association, 2013) and are one of the most common mental health disorders experienced by youth (Australian Institute of Health and Welfare [AIHW], 2014). Despite the sequelae of impairments, individuals with SUDs continue to use the substance/s leading to further dysfunction and neurobiological changes often expressed as persistent drug effects, including repeated relapses and intense drug craving (American Psychological Association, 2013). Additionally, excessive drug use and alcohol consumption can have debilitating acute and chronic effects on one’s physical and mental health (Das et al., 2016). Moreover, individuals with SUDs often remain engrained in destructive lifestyle patterns over time (Toumbourou et al., 2007), which may be exacerbated through comorbidities and co-occurring addictions.

## Substance Use Disorders in Youth: Prevalence, Risk Factors, and Implications

Substance use disorders are a pervasive public health concern, and notably, today’s youth are experiencing greater exposure to illicit drugs and alcohol than previous generations (Paglia-Boak et al., 2011). In Australia, it is estimated that 12% of youth (aged 12–17 years) have a SUD (Mission Australia, 2015), a prevalence mirrored in other developed countries (Crawford et al., 2015). In the United States, youth substance abuse has been labelled the single most significant public health problem, and one that has reached “epidemic proportion,” accounting for severe long-term health consequences and in excess of $65 billion (USD) in annual healthcare costs (National Center on Addiction and Substance Abuse, 2011). It is also noteworthy that the prevalence of SUDs in disadvantaged sub-populations may have been underestimated in existing reviews (Australian Institute of Health and Welfare [AIHW], 2014). These sub-populations include youth not attending school or no longer living at home, homeless and institutionalized people, and low socioeconomic populations – all of whom display higher substance use rates (Australian Institute of Health and Welfare [AIHW], 2014).

A concerning trend surrounding substance use is that the age of initiation for use of many types of illicit substances has decreased, with the age of initiation of first use falling to between 13 and 15 years (National Center on Addiction and Substance Abuse, 2011; Australian Institute of Health and Welfare [AIHW], 2014). Evidence also exists to indicate that earlier age-of-initiation contributes to a heightened risk of substance use later in adolescence and through adulthood (e.g., DeWit et al., 2000; Grant et al., 2001; Jordan and Andersen, 2016). Specifically, with each year that ‘age of substance use onset’ decreases, the odds of later substance abuse have been shown to increase by between 5 and 13.7% (DeWit et al., 2000; Grant et al., 2001).

The primary substance of misuse varies between individuals; however, alcohol, nicotine, marijuana (or cannabis), meth/amphetamines, opiates/pharmaceuticals, and ecstasy largely represent the most common substances of misuse (Australian Institute of Health and Welfare [AIHW], 2014; Mutter et al., 2015). The prevalence of youth using several substances at the same time (e.g., within the same month) is also increasing; such behavior is termed ‘poly-drug’ or ‘poly-substance’ use (Boys et al., 2001). Compared with adults, youth are more likely to be poly-drug users, and cite that such behavior is driven by the desire to ‘improve the effects’ of another drug, or to ‘help manage its negative effects’ (Boys et al., 2001). It was reported recently, for example, that abuse of two or more substances was present in approximately 56.5% of youth with SUDs (Mutter et al., 2015).

In cases where experimentation and risk-taking results in the development of SUDs in youth, it has been shown that these youth are less likely to obtain and maintain stable employment (Gray and Saggers, 2005), and are susceptible to health, behavioral, and interpersonal complications, including depression, anxiety, violence, trauma, post-traumatic stress disorder, suicidal thoughts, difficulties with schooling, negative leisure-time activities (stealing, drug use, loitering etc.), and family dysfunction (Prior et al., 2000; Ford et al., 2007; Staiger et al., 2009; Spanner, 2012). In the long-term, patterns of substance use that are established in youth appear relatively stable throughout adulthood (Toumbourou et al., 2007), and if not treated effectively, are associated with early mortality and morbidity (Toumbourou et al., 2007; Whiteford et al., 2013). The importance of identifying risk and protective factors, and their impact upon the progression of SUDs individuals, highlights the need for prevention and early intervention programs in youth.

## Contributors to Substance Use Disorders Among Youth

Youth exposure to illicit drugs and alcohol may occur (a) in utero, genetically, or through the toxicity of substances during gestation, (b) environmentally, through family and community influences (including parental substance abuse), and/or (c) through their own exploratory drug and alcohol use (Gilvarry, 2000). Although evidence indicates that genetic factors (e.g., gene-substance interactions) may predispose individuals to substance dependence (e.g., Baer et al., 2003), in this review we focus our attention on the *personal* and *environmental* factors (i.e., those that are more readily modifiable later in life) that may account for increased likelihood of SUD development. Our review of these issues is by no means exhaustive. Rather, our goal is to identify these risk (and protective) factors – and their impact upon the progression and persistence of SUDs – with the aim of highlighting the need for early and effective intervention programs in youth, and to help make the case for exercise participation as a viable adjunct treatment modality.

### ‘At-Risk’ Populations

The risk of substance misuse and SUDs, their consequences, and the processes for treatment and recovery, differ according to several factors, including gender, race and ethnicity, sexual orientation, age, culture, education, economic status, health status, and geographic location (e.g., Wang et al., 2005; Surís et al., 2008; Wisk and Weitzman, 2016; Vaeth et al., 2017). The major categories that classify a young person as ‘at-risk’ within Western societies – and therefore jeopardize the transition to healthy adult functioning – include (a) failure to complete Year/Grade 10, (b) unemployment or being in marginal or insecure employment, (c) engagement in behavior likely to bring oneself into the criminal justice system, (d) engagement in unsafe health practices, and (e) a family environment that fails to provide adequate safety and/or convey a sense of self-worth (Colthart, 1996). Minority groups are also significantly less likely to receive SUD treatment (Mutter et al., 2015), due to factors such as lower family income, lack of private health insurance, lack of support, language proficiency of parents, and racial/ethnic differences in stigma, attitudes, and cultural health beliefs (Cummings et al., 2011).

### Parents and Family

The family context is central in determining the development and persistence of SUDs, and plays an important role in shaping the success of treatment efforts (Lander et al., 2013). Infancy and the early developmental period is a time of substantial neurophysiological development arising out of the interactions between brain/mind/body of child and parent; optimally, this process results in the development of adaptative capabilities central to emotional self-regulation (Schore and Schore, 2007). In-turn, an individual’s ability to regulate their emotions is likely to be at least partially determined by their early attachment experiences (Flores, 2001). Close parent-child relationships, the provision of a secure attachment environment, and support manifested through affection, praise, and encouragement, provide a strong buffer against the likelihood of substance use (Knight et al., 1998; Flores, 2001; Schore and Schore, 2007). Conversely, negative influences during early developmental stages can have significant effects on brain development, emotional responses, and stress-coping strategies, with parent-child – particularly mother-adolescent – conflict a predictor of adolescent substance use (e.g., Farrell and White, 1998; Branstetter et al., 2011; Schore, 2017).

Branstetter et al. (2011) concluded that greater support within mother–child relationships was associated with less frequent use of all substances in the 10th and 11th grade, as well as fewer negative behavioral outcomes (e.g., negative interactions with peers, negative parental interactions, concurrent substance use or hard drug use). Additionally, it has been shown that a low level of perceived father support is associated with an increased likelihood of all types of substance use (Piko, 2000). These early relational experiences play a key role in the development of both psychological and neurological pathways, and contribute to fostering either secure or insecure attachments that carry neurobiological implications throughout the lifespan (Schore and Schore, 2007; Schore, 2017).

Other family-related factors that are associated with an increased risk of youth substance abuse include unclear expectations for behavior, inconsistent family management practices, lack of (or inconsistent) discipline, low parental educational aspirations, poor monitoring of behaviors, excessive punishment, a hostile environment, various forms of abuse (e.g., verbal, physical, and sexual), and family conflict (Van Der Vorst, 2012; Berge et al., 2016). Moreover, given the heritability of addictions (40–60%), and in light of behavioral evidence demonstrating that youth tend to mirror patterns of parental or family substance use (Derringer et al., 2008), it has been demonstrated that youth of substance-using parents are at heightened risk of initiating substance use at an earlier age, developing a SUD or substance dependence, and experiencing mental health disorders (Kilpatrick et al., 2000; Koob and Volkow, 2016; Zehetner et al., 2016).

### Peers

Peer groups, and interactions with one’s peers, play an important role in shaping the likelihood of both initiating and maintaining substance use and/or abuse patterns (e.g., Piko, 2000; Iwamoto and Smiler, 2013; Studer et al., 2014). Substance use among one’s peers has been reported to be the most consistent positive predictor of adolescents’ substance use over time (Branstetter et al., 2011). Peers can provide immediate access to substances, influence one’s normative perceptions about the legitimacy of substance use, model drug using behavior, and help shape beliefs and attitudes toward substance use (e.g., Farrell and White, 1998; Branstetter et al., 2011). Consequently, the availability of substances through one’s peers, and a decrease in perceived risk associated with the use of illicit drugs may contribute to the peer-related effects on substance use (Australian Institute of Health and Welfare [AIHW], 2014).

The desire to conform to one’s peers is particularly heightened during adolescence (Coleman and Hendry, 1990), and although this desire may engender positive outcomes when peers model ‘desirable’ or healthy behavior, it may also come at the cost of encouraging unhealthy or risk-taking behavior (e.g., substance use) that may not be consistent with one’s own views or principles (Iwamoto and Smiler, 2013). In addition to conformity pressures, youth may also experience significant concerns relating to peer rejection and defiant friendship networks, both of which may also be predictors of antisocial behavior and substance use (Spanner, 2012).

### School

Low achievement at school, negative school experiences, adolescents’ beliefs about school, and their academic expectations all represent risk factors for substance misuse (e.g., Bryant et al., 2003). School misbehavior is indicated to be positively associated with substance use during adolescence, and it is also recognized that misbehavior in school may transfer to other settings (e.g., with peers in social situations) and provide adolescents with more opportunities to engage in substance use (Bryant et al., 2003). Importantly, schools are recognized as important sites for SUD prevention efforts in terms of health promotion, social support, setting behavioral norms, and establishing guidelines for student behavior control (Evans-Whipp et al., 2004; Das et al., 2016).

### Stress

Stress plays a significant role in contributing to substance use (and relapse from substance use) among vulnerable youth (Sinha, 2001). It is proposed that stress negatively impacts individuals through alterations in abilities to generate and execute effective decisions and behaviors (Fishbein et al., 2006). Stressful and traumatic experiences early in life may increase one’s vulnerability to drug use (Sinha, 2001; Koob and Volkow, 2016), and higher levels of stress in youth are positively associated with alcohol consumption, and nicotine and marijuana use (e.g., Kaplan and Johnson, 1992; Wills et al., 1996; Sinha, 2001). Furthermore, engaging in alcohol and drug use as a coping mechanism in response to stress is positively associated with dependence symptoms and compulsive drug use in adolescents (Laurent et al., 1997). Additionally, there is evidence that drug exposure and chronic use may drive stress-like states and increased stress responsiveness as a result of neurobiological and developmental changes that are compounded by decreases in ‘anti-stress’ mechanisms (Koob and Simon, 2009; Koob and Volkow, 2016). The multifactorial processes underlying the proposed stress surfeits are complex and not fully understood, however, they are notable given high-stress system functioning is coupled with reward deficits that drives compulsive use and pathological drug seeking (Koob and Volkow, 2016).

### Boredom

Adolescence – and the transition from being dependent on one’s parents to forming autonomous friendship groups – is marked by an increase in leisure-time and greater amounts of time spent with peers (Barnes et al., 2007). As a correlate (in some instances) of the greater leisure-time that adolescents experience, boredom may prevail, and boredom has been cited as a primary reason as to why youth engage in substance use and later may subsequently develop SUDs, as well as relapse to use following a period of abstinence (e.g., Levy, 2008; Sharp et al., 2011; Dow and Kelly, 2013). At an individual level, boredom may encompass a variety of elements from not knowing what to do with one’s time, through to an emptiness associated with social isolation, or a lack of attachment and relatedness to others (Levy, 2008). Regardless of the underlying cause, however, boredom is associated with poorer youth outcomes and is a critical factor in relapse (Levy, 2008). Youth who report high levels of boredom tend to display greater involvement in risk-taking behaviors, extreme sensation activities and/or various forms of delinquency in an attempt to combat that boredom (Patterson et al., 2000). Boys et al. (2001) reported that up to 88.5% of youth (16–22 years) poly-substance users relied on illicit drugs to enhance an activity, and 83% of this sample engaged in substance use to decrease boredom. Moreover, feelings of boredom have been cited to be extremely prevalent when individuals initially cease substance use, thus contributing to a heightened risk of relapse (e.g., Powers, 2007; Levy, 2008). For many individuals, substance use is used to occupy one’s time in the absence of ‘healthy’ interests and leisure activities (Levy, 2008), and that being the case, youth in recovery from SUDs may benefit from engaging in (healthy) alternative activities that reduce the tendency for boredom.

### Mental Health

Comorbidity of SUDs and other mental health disorders is common (Gordon, 2009), with comorbid conduct disorder, oppositional disorder, and/or depression suggested to be highly prevalent among youth with SUDs (Conway et al., 2016). Researchers examining the association between comorbid mental health disorders and SUDs have revealed that 64% of substance abusing youth across 23 different youth treatment programs (residential, short-term inpatient, outpatient drug-free) had at least one comorbid mental disorder; 59% were diagnosed with conduct disorder, 15% with depression, and 13% with attention deficit hyperactivity disorder (Grella et al., 2001). In other reports, it has been documented that up to 93% of youth with a SUD experience co-occurring mental health disorders (Lichtenstein et al., 2010), and Conway et al. (2016) highlighted that alcohol and drug abuse was highest among youth with prior anxiety disorders and behavior disorders relative to youth with no mental health disorders. It is also possible that, in some instances, suboptimal mental health may mediate the effects of other personal and environmental factors (e.g., poor family support, stress) on SUD development and persistence, and so it is important to consider intervention strategies that target these factors as well as seeking to improve mental health.

## Current Treatment Methods

The prevalence of SUDs in youth, and the risks associated with persistent substance use during this developmental period, highlight the need for effective treatment interventions that support abstinence and harm minimisation (Marlatt and Witkiewitz, 2002; Evans et al., 2005; Dow and Kelly, 2013). Many studies with adult populations have demonstrated feasibility and effectiveness of SUD treatment programs; however, no consistent and universally accepted approach exists (see, for example, Glasner-Edwards and Rawson, 2010; Sobell et al., 2013). In youth, treatment options are not as well developed, with statistics from the United States indicating that only 7–10% of youth in need of SUD treatment actually receive care (Mutter et al., 2015) and similar trends exist in Australia (Reavley et al., 2010). In addition, treatments targeting SUDs in youth are commonly based on adult SUD strategies, with limited attention devoted to how treatment may be best tailored for youth (Gilvarry, 2000; Mutter et al., 2015).

Treatment modalities, their duration and stages of implementation vary (e.g., Dutra et al., 2008), however, the primary goal of many SUD rehabilitation programs is abstinence, attained through the treatment of the physiological, psychological, and sociological problems presented by the individual (e.g., Centre for Substance Abuse Treatment, 1997; Sher, 2014). Abstinence, however, is not the only (or primary) goal in some instances/programs, with some indication that the goal of abstinence may be ineffective in reducing substance use and abuse (Marlatt and Witkiewitz, 2002). More holistic goals, including harm minimisation, facilitating access to education, reducing substance use, improving interpersonal relationships, and improving physical and mental health, may also be targeted (Fisher and Roget, 2009; Marsh et al., 2013).

Compared to adults, youth are more likely to be referred to SUD treatment facilities by the criminal justice system, with an estimated 44.5% of youth with SUD referred via this pathway (Mutter et al., 2015). The criminal justice system referral pathway may influence motivation to change one’s substance using behavior (Winters et al., 2007), either acting as an external motivation to avoid readmission, or a barrier to effective treatment since youth may feel ‘forced’ to complete the treatment (Ryan and Deci, 2008). Other common avenues for referral, or accessing treatment, include self-referral (including family referral), community referral (including Federal, State or local agencies; e.g., Child Protective Services, homeless shelters), and health care provider referral (e.g., physicians, psychologist, mental health program) (Mutter et al., 2015). Many additional challenges – beyond those associated with the method of entry – exist in treatment, with the primary challenge being that youth are characterized by broad differences in recovery motivation, substance involvement and impairment, and psychological comorbidities (Dow and Kelly, 2013). However, for logistical and financial reasons, the majority of treatment is delivered in group formats (Velasquez et al., 2015), making it more challenging to tailor treatment to the personal needs of youth with SUDs.

Co-occurring physiological, psychological, and sociological factors heighten the difficulty of SUD recovery. Substance use relapse is a primary concern throughout SUD treatment, and youth-based literature predominantly reports low rates of continuous abstinence following treatment (Chung and Maisto, 2006). Definitions of substance relapse vary, ranging from a single occasion of substance use, or ‘lapse,’ to returning to problematic ongoing use (Chung and Maisto, 2006). Gonzales et al. (2012) reported, for instance, that approximately 65% of youth relapse in the first 90 days following treatment, with this rate increasing to about 85% in the 12 months following treatment. Reasons for relapse are diverse, but may include social pressure, withdrawals, negative affect, interpersonal conflict, the presence of co-occurring psychological symptoms and a lack of perceived importance toward remaining abstinent (e.g., Cornelius et al., 2003; Ramo et al., 2005; Chung and Maisto, 2006; Ramo and Brown, 2008). Unfortunately, rapid relapse to substance use is often considered the norm among adolescents who have completed a period of SUD treatment (Cornelius et al., 2003). As a result, in addition to evaluating the effectiveness of current treatment strategies, it appears important that we search for treatment approaches that may assist in breaking the addiction-recovery-relapse cycle.

## Does Exercise Help in the Treatment of Substance Use Disorders? Evidence From Adult Populations

Adopting a multidisciplinary approach to SUD rehabilitation that incorporates structured exercise participation may offer a novel and effective complement to current treatment approaches. In particular, it is recognized that exercise participation may (a) help to alleviate a number of the factors that contribute to SUD development and that act as barriers to healthy recovery (e.g., a lack of social support, poor mental health, high stress, and boredom), and (b) support the ‘holistic’ goals that are pursued within some treatment programs (e.g., improving interpersonal relationships, and physical and mental health). For example, exercise participation not only improves mental health (Penedo and Dahn, 2005; Stathopoulou et al., 2006), it also alleviates stress (Pedersen and Saltin, 2015), provides stimulation and an outlet for boredom (Sherwood and Jeffery, 2000; Martin et al., 2006), may contribute to more positive social interactions (Smith, 2003; Read et al., 2004), and can be delivered in the group-based format that is typically adopted in existing treatment programs (Velasquez et al., 2015). Moreover, exercise participation – such as during residential SUD treatment – may help substance users develop a routine and better utilize their leisure time (Spanner, 2012). In doing so, exercise participation may provide a viable alternative to recreational activities that involve illicit substances (Elder et al., 2000), and contribute to the development of physical activity patterns that facilitate a less traumatic transition from care into the community (see Weinstock et al., 2012).

There is a dearth of research evidence to support the role of exercise as an adjunct treatment for SUDs in youth; however, there is support for the beneficial effects of this type of therapy in adult SUD populations. In terms of treatment outcomes, studies have shown positive effects of exercise interventions for reducing alcohol consumption (e.g., Brown et al., 2009, 2014; Hallgren et al., 2014), reducing nicotine and illicit drug use (e.g., Bardo and Compton, 2015; Muller and Clausen, 2015), improving abstinence (e.g., Wang et al., 2014; Brellenthin and Koltyn, 2016; De La Garza et al., 2016), and reducing the urge to drink (Hallgren et al., 2014). Improvements in physical fitness have also been reported (e.g., Dolezal et al., 2013; Giesen et al., 2015; Hallgren et al., 2017), as have adaptive psychological outcomes in the form of improved well-being and sleep quality (e.g., Hallgren et al., 2014), reduced depression (see, for example, Stathopoulou et al., 2006; Roessler et al., 2013; Hallgren et al., 2017), reduced anxiety (see Wang et al., 2014; Giesen et al., 2015), improved overall mental health (Penedo and Dahn, 2005), and elevated quality of life, mood, and motivation (e.g., Ciccolo et al., 2015; Muller and Clausen, 2015). Also, in terms of relapse prevention, researchers have demonstrated that in early recovery, the benefits of exercise may include improved mood, decreased urges and cravings, increased self-efficacy for abstinence, and increased function as a useful coping strategy (Buchowski et al., 2011; Brown et al., 2014).

Modality, duration, and intensity of exercise interventions across the adult SUD literature are varied, with no consistent approach that has demonstrated the greatest feasibility or benefit. For example, combined aerobic and resistance, solely aerobic, exclusively resistance exercise, and yoga-based programs have all been successfully implemented, solely aerobic, exclusively resistance exercise, and yoga-based programs have all been successfully implemented (see, for example, Roessler et al., 2013; Wang et al., 2014). Intensity of exercise also varies from low- to high-intensity activity, and in terms of program duration, evidence exists to support acute benefits, as well as 6-, 8-, and 12-week program outcomes (e.g., Roessler et al., 2013; Flemmen et al., 2014; Hallgren et al., 2014; Wang et al., 2014). In light of the effectiveness of exercise interventions in adult SUD populations, it appears plausible that similar benefits may be achieved among youth populations recovering from a SUD.

Examining exercise attitudes, beliefs, and preferences of adults with SUDs may also inform us about how best to structure exercise within a holistic, multidisciplinary treatment approach to SUDs in youth. Stoutenberg et al. (2015) examined individuals’ attitudes and preferences toward exercise training in adults exclusively with alcohol use disorder in a residential treatment setting (Stoutenberg et al., 2015). Surveys administered within 2 days of intake into the treatment center revealed that approximately 70% of respondents were in favor of receiving exercise training as part of their treatment, with 90% preferring a face-to-face format, and 76.5% of respondents preferring a variety of exercise modalities (Stoutenberg et al., 2015). An earlier study among adults with alcohol use disorder supported these findings, concluding that the majority (54%) of participants reported they would be ‘very’ or ‘extremely’ interested in participating in an exercise program (a further 21% indicated a ‘slight’ or ‘moderate’ interest) (Read et al., 2001).

Similar positive attitudes have been reported among adults with SUDs, with 95% of patients in an intensive substance abuse outpatient program reporting an interest in engaging in a specifically designed exercise program (Abrantes et al., 2011). A large proportion (i.e., 89%) of individuals also expressed a desire to commence exercise within the first 3-months of sobriety, or in early recovery (Abrantes et al., 2011). This pattern is also supported in literature in which attitudes and experiences of heroin users have been examined (Neale et al., 2012). Adult heroin users, either starting a new episode of drug treatment or who had recently ceased using, expressed through focus group interviews that they were ‘very interested’ in engaging in sport and exercise (Neale et al., 2012). Moreover, Neale et al. (2012) reported that deriving enjoyment was as a key feature associated with the desire to be physically active in treatment and early recovery. Although much less is known about youth’s attitudes toward exercise participation within SUD treatment, the consistently positive attitudes that have been reported among adults do appear to support the inclusion of adjunct exercise programs within youth SUD treatment programs.

In addition to documenting individuals’ attitudes to exercise within adult SUD treatment facilities, perceptions about barriers to, and preferences for, exercise participation have also been identified. Researchers have reported that perceived barriers to exercise participation among adults with SUDs include ‘not being able to afford it,’ having ‘no access to equipment,’ ‘lack of motivation to get started,’ ‘lack of knowledge,’ ‘lack of confidence,’ ‘transportation,’ ‘not having the energy,’ ‘not being able to keep up,’ ‘feeling uncomfortable exercising,’ and, ‘not having anyone to do it with’ (Abrantes et al., 2011; Neale et al., 2012; Stoutenberg et al., 2015). In terms of health-related challenges, ‘heavy drug use,’ ‘poor health,’ and ‘psychological issues’ have also been cited as barriers (Neale et al., 2012). These barriers did not differ according to treatment type, and interestingly, a ‘lack of time’ was not identified as a barrier, which is one of the most commonly cited barriers in non-SUD populations (Borodulin et al., 2016). Preferred modality of exercise varies in the literature; walking and strength/resistance training appear to represent the most preferred activities, and intensity preferences vary from moderate to higher intensity exercise (Abrantes et al., 2011; Stoutenberg et al., 2015). Clearly, the variation in terms of exercise modality preferences highlights the need to develop exercise interventions in consultation with program participants. Moreover, the barriers outlined above also inform the way in which exercise programs should be structured so as to minimize challenges that participants may face (i.e., programs should focus on low-cost, self-paced exercise options that are not reliant on excessive transportation, as well as targeting social support provision and confidence-enhancement). Notwithstanding these considerations, it is recognized that exercise interventions can provide effective, low-cost adjunct therapies with numerous secondary health benefits within SUD treatment facilities (Abrantes et al., 2011).

## Can Exercise Help Youth with Substance Use Disorders?

Although knowledge of the outcomes of exercise participation within youth SUD populations is limited, there is a significant body of research within non-SUD youth populations that demonstrates the short- and long-term benefits of regular exercise. For example, in an acute sense, exercise contributes to improved physical fitness and enhanced emotional state, including reduced depression and anxiety symptoms (Penedo and Dahn, 2005; Puetz et al., 2006). In the longer-term, evidence exists for the effectiveness of regular exercise in the prevention of multiple chronic diseases (e.g., diabetes, hypertension, obesity, osteoporosis, cardiovascular disease, and cancer) and premature death (Penedo and Dahn, 2005; Warburton et al., 2006). Within youth populations, regular exercise stimulates increased energy, maintenance of a healthy weight, prevention of osteoporosis, some cancers and heart disease later in life, and improved academic performance (Ströhle et al., 2007; Biddle and Asare, 2011). Additionally, exercise has positive mental health properties for youth, including reduced depression and anxiety, and improved self-esteem (Ströhle et al., 2007; Biddle and Asare, 2011). With particular relevance for this review, Ströhle et al. (2007) – when presenting the results of a cross-sectional study with a community cohort of youth aged 14–24 years – concluded that regular exercise was associated with a decreased prevalence of co-morbid mental health disorders, due to lower rates of SUDs, anxiety disorders, and dysthymia. Ströhle et al.’s findings mirror those reported in other investigations of cross-sectional associations between youth exercise (or sport) participation and substance use, in which it has been demonstrated that greater exercise or sport engagement aligns with reduced substance (e.g., marijuana, alcohol, and cocaine) use (e.g., Pate et al., 2000; Terry-McElrath et al., 2011). Furthermore, Brosnahan et al. (2004) investigated self-reported ‘feelings of sadness and hopelessness’ as apparent synonyms for depression alongside ‘suicidal thoughts and behaviors’ in youth, with results suggesting exercise participation may account for the alleviation of these feelings. Considering the wide-ranging benefits associated with exercise participation among youth – and in particular those outcomes that align with SUD risk factors (e.g., mental well-being, stress alleviation) – it is possible that regular exercise participation within youth SUD treatment programs may contribute to more effective rehabilitation and support mental health and abstinence outcomes.

To date though, very little research attention has been directed to this important issue, and as a result, the feasibility and/or outcomes of exercise participation within youth SUD populations are relatively unknown. In one of the few examples of research in this area, an unpublished thesis investigated the experiences of eight male youth (15–21-years) in a residential addiction treatment program that included exercise and leisure education (Spanner, 2012). Recruited from an inpatient addiction treatment facility, participants in this study undertook a 4-week intervention consisting of aerobic and resistance training, leisure education, and pre- and post-program interviews. Participants reported mental health-related benefits associated with exercise, including increased self-confidence and concentration, positive variation in mood, and improvements in energy. Additionally, youth in this study described noticing improvements in their physical health, placing greater value on their health, an increased motivation to exercise, and reduced negative feelings toward exercise. Although the modest sample size and male-only population limits the generalisability and scope of these findings, the results of this study do offer encouragement for the inclusion of exercise within youth SUD treatment programs.

Collingwood et al. (1991) were among the first to support the inclusion of structured exercise programs for youth with SUDs. In their study, 74 adolescents (with an average age of 16.8-years) in a school-based ‘at-risk’ prevention program, a community counseling agency substance abuse program, or an in-patient hospital-based drug intervention program, took part in a 9-week structured physical fitness group program comprising aerobic exercise and strength development. Participants engaged in one or two, 1.5-h class/es per week and were encouraged to complete two additional individual exercise sessions per week on their own or with a peer. Encouragingly, participants demonstrated significant increases in self-concept and physical measures (i.e., field fitness tests including one mile run, 1 min sit up, 1 min push-up, sit and reach flexibility test, skinfold body fat test), decreases in anxiety and depression risk factors, and significantly lower self-reported substance use patterns (i.e., higher abstinence; Collingwood et al., 1991). It is important to note that this work focused only on the moderate-to-long-term outcomes of exercise on youth with SUDs, and did not offer evidence for the impact of exercise in the acute, or detoxification, stage of rehabilitation. Moreover, youth were also attending various rehabilitation facilities, and therefore varied in terms of substance dependence or addiction. However, the design (e.g., including peer support) and outcomes of this study do offer insight into how to optimize exercise programs within SUD treatment. It is unfortunate, therefore, that – since the publication of this encouraging work – sustained research effort has not been directed toward examining the mechanisms through which exercise may support recovery and mental health outcomes among youth with SUDs.

Despite the lack of research examining exercise as an adjunct treatment method for youth with SUDs, evidence does exist to support its use in other forms of addiction, as well as within ‘at-risk’ or ‘delinquent’ youth. As is the case with SUDs, youth smoking tends to predict adult smoking, and leads to an increased risk of long-term nicotine and alcohol dependence (Everson et al., 2006). Following a 10-week exercise-plus-education cessation intervention, Horn et al. (2011) reported that youth with nicotine addiction displayed higher smoking cessation rates compared to an education-only control group, and these cessation rates were also maintained at a 6-month follow up. Similarly, Everson et al. (2006) concluded that, following an acute 10-min bout of moderate intensity exercise, participants’ desire to smoke and withdrawal symptoms were reduced, and affective responses over time were improved. Despite a lack of specific details on the sample (e.g., substance used and diagnosis), earlier work by Collingwood (1972) also supports these benefits. In particular, males aged 18–26 who attended a rehabilitation facility reported increases in positive self-attitudes and self-acceptance, and positive physical, intellectual, and emotional-interpersonal behaviors following a structured 1-h, 5-days-a-week, 4-week exercise program including endurance and cardiovascular work, strength exercises and agility drills. In another study, Hilyer et al. (1982) assessed the effect of physical fitness training among youth offenders. In this work, 60 youth within a state industrial school for youth considered to be high security risk engaged in a 1.5-h, 3-days-a-week, 20-week physical fitness program consisting of cardiorespiratory, strength, endurance and flexibility training. Following the intervention, it was reported that youth had elevated self-esteem, reduced anxiety and depression, and a healthier psychological state. Although the number of studies that have examined exercise outcomes among youth in SUD treatment are limited, the consistent findings within these studies – with respect to recovery and a range of mental health indices – appear to provide compelling evidence for the efficacy of exercise interventions as adjunct treatment for youth with SUDs.

## Recommendations for Future Research

There appears to be sufficient support for the notion that exercise interventions may contribute to successful SUD recovery among youth. At this point, though, little is known about the feasibility and implementation of such approaches, and large-scale controlled trials that quantify the outcomes associated with these strategies have yet to be conducted. Below, we present some key recommendations for research that we hope will advance our knowledge about the inclusion of exercise programs within youth SUD treatment. In doing so, we acknowledge that these recommendations are by no means exhaustive, and encourage researchers to consider issues beyond those detailed below.

### Recommendation 1: Examine Exercise Perceptions and Attitudes among Youth with SUDs

An important first step for research in this area is to establish the feasibility of incorporating tailored exercise programs as an adjunct therapy for youth recovering from a SUD. Accordingly, assessing (a) youth’s perceptions and attitudes toward exercise involvement (e.g., how is exercise participation considered and received by youth undergoing SUD rehabilitation), (b) perceived barriers to exercise participation (i.e., the factors that hinder or challenge exercise involvement), and (c) facilitators of exercise participation (i.e., things that encourage exercise involvement) is a vital first step in devising effective exercise programs for this population. Additionally, it is important that we seek to better understand youth’s preferences regarding exercise modality, intensity, and frequency, as well as the ways in which exercise might best be integrated into recovery treatment (e.g., by using exercise facilities within a treatment center, by creating links with community sport/exercise organizations, etc.). In light of the findings reported previously regarding adults’ (positive) perceptions about the value of exercise participation alongside SUD treatment (e.g., Abrantes et al., 2011; Stoutenberg et al., 2015), it is likely that the majority of youth with SUDs would consider exercise a positive addition to traditional rehabilitation procedures, and that barriers toward exercise participation would reflect substance-related (e.g., cravings, withdrawals), mental health (e.g., mood), and physical health (e.g., injury) factors. This formative work could be conducted without the need for actual exercise participation/programs, and in terms of design considerations, in-depth interviews and focus groups that include youth with SUDs as well as other relevant stakeholders and policy-makers (e.g., staff at recovery treatment centers, exercise practitioners, allied health service providers) would be worthwhile. From a research perspective, this information would be valuable for the development of successful exercise programs and ensuring participant motivation in further work. Meanwhile, in a practical sense, the findings from this work would also inform allied health professionals about how best to deliver exercise programs in both residential and community settings for youth recovering from SUDs.

### Recommendation 2: Examine Recovery-Related Outcomes Associated with Exercise Participation

In seeking to better understand (and build an evidence base relating to) the value of exercise as adjunct therapy, testing the influence of exercise participation on recovery-specific and mental health outcomes – both in the short-term and in the longer-term – is crucial. With that in mind, researchers are encouraged to develop suitably powered cluster randomized trials that examine the effectiveness of treatment programs involving exercise participation relative to matched, no-exercise (e.g., standard care) controls. In line with the goals of harm minimisation and abstinence-based models of treatment (Marlatt and Witkiewitz, 2002; Marsh et al., 2013), it is important to assess a range of relevant recovery-related outcomes (e.g., abstinence behavior/thoughts, cravings and withdrawals), as well as mental health indices (e.g., self-esteem, mood, perceived support, coping skills) and relational factors (e.g., family interactions, peer/interpersonal relationships) – these mental health and relational processes may act as mediators of recovery effects and/or as important outcomes in their own right. Attention should also be paid to best-practice recommendations during the design and conduct of such studies (Schulz et al., 2010), and sample size (and study design) considerations may need to account for treatment and exercise program attrition (for information on potential dropout rates, see Muller and Clausen, 2015). Guided by the findings within the limited number of studies that have assessed exercise participation in youth SUD treatment (Collingwood et al., 1991; Spanner, 2012), and the larger literature base relating to the use of these strategies within adult SUD treatment (e.g., Brown et al., 2014; Ciccolo et al., 2015; Brellenthin and Koltyn, 2016; De La Garza et al., 2016), it might be hypothesized within these trials that exercise participation would account for adaptive recovery outcomes, and engender positive effects upon mental health and interpersonal relationships. On a related note, although we have directed much of our attention (and many of our recommendations) toward the role of exercise for youth within residential treatment programs, there are other well-established intervention modalities that would also benefit from investigations into the feasibility and efficacy of exercise programs. Family-based therapy, for example, is a recognized treatment approach for adolescent substance use (e.g., Liddle et al., 2005; Horigian et al., 2016), and the implementation of (and assessment of recovery outcomes derived from) family-based exercise interventions within these settings would be extremely valuable.

In addition to addressing the recovery-specific (and other health) outcomes, it would also be of value to investigate the potential role of exercise on SUD rehabilitation compliance. Relapse throughout treatment is largely considered the norm among youth, and relapse rates are higher than rates of successful completion (Cornelius et al., 2003; Ramo et al., 2005; Ramo and Brown, 2008). Exercise participation may address a number of the pathways that heighten the likelihood (and persistence) of youth SUDs (e.g., alleviating boredom and stress, improving peer relationships and mental health), and if exercise is successful in achieving these effects *during* treatment in residential centers (or, for example, in family-based settings), exercise may indirectly (through these effects) also contribute to greater rehabilitation compliance and increased rates of treatment completion. The potential for such outcomes, to our knowledge, has yet to be addressed within the literature; however, there may be important public health and economic implications should exercise be shown to contribute to improved rehabilitation compliance.

### Recommendation 3: Identify Community Transition Pathways and Long-Term Outcomes

Long-term abstinence, harm minimisation, and the maintenance of a healthy lifestyle represent the overarching goals of SUD treatment (Marlatt and Witkiewitz, 2002; Marsh et al., 2013). Effective therapy and treatment programs, therefore, should assist individuals in reaching these goals. In our previous recommendations for research, we have considered the feasibility and efficacy of exercise participation *during* one’s treatment (e.g., when in residential care). However, although exercise participation for individuals within residential SUD treatment may be feasible (given that these individuals are in care, are supervised, and may have access to facilities), the extent to which exercise participation can be maintained when transitioning back into the community is largely unknown. That being the case, researchers are encouraged to examine whether, and how, exercise participation might be promoted not only during one’s treatment, but also *following* the completion of treatment. In doing so, it would be advisable to follow the same general approach outlined above by (a) first gaining an understanding of the various challenges and barriers that youth may face in terms of maintaining exercise participation when in the community, and (b) examining whether the introduction of behavior change techniques – such as planning, self-monitoring, and enlisting support (see, for example, Michie et al., 2011) – during and following one’s treatment might support more successful maintenance of exercise participation.

The factors that contribute to an increased likelihood of youth developing SUDs are well established. With that in mind, and notwithstanding the significant challenges inherent in such work, researchers might also be encouraged to consider these factors (e.g., ‘at-risk’ youth, and those with a history of family conflict, poor mental health, low academic achievement, high stress, etc.) when targeting populations for community-based exercise promotion efforts. Although we have focused on the potential ‘curative’ properties of exercise for youth who have SUDs (i.e., providing exercise as a treatment strategy), it is possible that the various benefits accrued through regular exercise may also make it viable for the *prevention* (at least in part) of SUDs among ‘at-risk’ youth. Successful community exercise and physical activity programs that are targeted at these ‘at-risk’ individuals may play a protective role in reducing the likelihood of SUD onset and/or limiting the severity of SUDs in instances when they begin to develop; research that tests this proposition would be extremely valuable.

## Summary

Substance use disorders are one of the most common and debilitating mental health conditions experienced by youth, and several well-established risk factors exist that contribute to the development and persistence of SUDs. Importantly, patterns of substance use that are established in youth appear to remain relatively stable throughout one’s life, highlighting that a search for more effective treatment modalities is needed in order to support more effective (and lasting) recovery outcomes. Incorporating structured exercise participation within SUD rehabilitation programs has been shown to be feasible in adult populations, and appears to stimulate more effective recovery outcomes as well as physical and mental health benefits. To date though, despite the well-established health outcomes associated with exercise participation, there is little evidence for the role of exercise as a complement to standard SUD treatment among youth. We encourage researchers and practitioners to consider this opportunity to develop what is known regarding youth SUD treatment, and to contribute to advancing our understanding about how exercise participation may underpin successful SUD treatment and prevention.

## Author Contributions

All authors listed have made a substantial, direct and intellectual contribution to the work, and approved it for publication.

## Conflict of Interest Statement

The authors declare that the research was conducted in the absence of any commercial or financial relationships that could be construed as a potential conflict of interest.
